# Relevance of *XPD* polymorphisms to neuroblastoma risk in Chinese children: a four-center case-control study

**DOI:** 10.18632/aging.101522

**Published:** 2018-08-08

**Authors:** Jiwen Cheng, Zhenjian Zhuo, Yijuan Xin, Pu Zhao, Weili Yang, Haixia Zhou, Jiao Zhang, Ya Gao, Jing He, Peng Li

**Affiliations:** 1 Department of Pediatric Surgery, the Second Affiliated Hospital of Xi'an Jiaotong University, Xi'an 710004, Shaanxi, China; 2 School of Chinese Medicine, Faculty of Medicine, The Chinese University of Hong Kong, Hong Kong 999077, China; 3 Clinical Laboratory Medicine Center of PLA, Xijing Hospital, Air Force Medical University, Xi'an 710032, Shaanxi, China; 4 Department of Neonatology, Shaanxi Provincial People's Hospital, Xi'an 710068, Shaanxi, China; 5 Department of Hematology, The Second Affiliated Hospital and Yuying Children's Hospital of Wenzhou Medical University, Wenzhou 325027, Zhejiang, China; 6 Department of Pediatric Surgery, the First Affiliated Hospital of Zhengzhou University, Zhengzhou 450052, Henan, China; 7 Department of Pediatric Surgery, Guangzhou Institute of Pediatrics, Guangzhou Women and Children’s Medical Center, Guangzhou Medical University, Guangzhou 510623, Guangdong, China; * Equal contribution

**Keywords:** neuroblastoma, *XPD*, polymorphism, susceptibility, DNA repair

## Abstract

Neuroblastoma is a lethal tumor that commonly occurs in children. Polymorphisms in *XPD* reportedly influence risk for several types of cancer, though their roles in neuroblastoma remain unclear. Here we endeavored to determine the relevance of *XPD* gene polymorphisms and neuroblastoma susceptibility in Chinese children genotyping three *XPD* polymorphisms (rs3810366, rs13181 and rs238406) in 505 cases and 1070 controls and assessing their contributions to neuroblastoma risk. Overall, we detected no significant association between any single *XPD* genotype and neuroblastoma risk. When risk genotypes were combined, however, we found that patients with 2-3 risk genotypes were more likely to develop neuroblastoma (adjusted odds ratio =1.31; 95% confidence interval =1.06-1.62, *P*=0.013) than those with 0-1 risk genotypes. Stratification analysis of rs3810366 revealed significant relationships between the subgroups age ≤18 months and clinical stage I+II+4s and neuroblastoma risk. Moreover, the presence of 2-3 risk genotypes was significantly associated with increased neuroblastoma risk in the subgroups age ≤18 months, male, tumor originated from others, and clinical stage I+II+4s. Our findings provide novel insight into the genetic underpinnings of neuroblastoma and demonstrate that *XPD* polymorphisms may have a cumulative effect on neuroblastoma risk.

## Introduction

Neuroblastoma, a solid tumor of the sympathetic nervous system, remains the most commonly occurring lethal cancer among children [1], accounting for 7%-10% of all cancers in children under 15 years of age [2,3]. The prevalence of neuroblastoma in the United States is about 1 in 7000 [4], but is only about 1 in 13,000 in China [5]. Neuroblastomas have been classified into low-risk, intermediate-risk and high-risk groups [6]. Among them, the high-risk group accounts for 50% of all neuroblastoma patients. The 5-year survival rate among this high-risk group remains less than 35%, despite administration of intense multimodal therapy [7,8]. This low cure rate may be attributable to the presence of widespread tumor metastasis at the time of diagnosis [9].

About 1% of neuroblastoma patients present with hereditary disease [10]. The etiology of familial neuroblastoma is mainly explained by germline mutations in *PHOX2B* [11,12] and *ALK* [13,14]. Although, there is as yet no explicit description of the causes of sporadic neuroblastoma, there is growing evidence suggesting genetic and genetic-environmental factors affect ones susceptibility to neuroblastoma [15,16]. Recent genome-wide association studies identified DNA alleles that contribute significantly to the risk of neuroblastoma, including *BARD1* [17], *TP53* [18], *HACE1* [19], *LIN28B* [19], and *MMP20* [20]. Moreover, candidate gene approaches also associated *NEFL* [21] and *CDKN1B* [22] polymorphisms with neuroblastoma susceptibility.

The integrity and stability of the human genome is maintained primarily by DNA repair systems [23], among which the nucleotide excision repair (NER) pathway is essential in eliminating DNA damage caused by both exogenous and endogenous factors [24]. Dysfunction of the NER pathway could lead to failure to repair genomic defects, thereby increasing cancer risk [25–27]. Several critical genes (*ERCC1*, *XPA*, *XPB*, *XPC*, *XPD*, *XPE*, *XPF* and *XPG*) participate in the NER process and coordinately function to maintain genomic integrity [28]. *XPD* (xeroderma pigmentosum complementary group D), also known as *ERCC2* (excision repair cross-complementation group 2), encodes an evolutionarily conserved ATP-dependent helicase [29]. This helicase forms a complex with the transcription factor TFIIH to function in basal transcription and nucleotide excision repair [30]. The importance of *XPD* to the NER pathway has prompted several case-control studies to assess the effect of *XPD* polymorphisms on the risk of such cancers as nasopharyngeal carcinoma [31], renal cell carcinoma [32], prostate cancer [33], esophageal squamous cell carcinoma [34], and breast cancer [35]. However, the effect of *XPD* polymorphisms on neuroblastoma risk has not yet been studied. To address that issue, we conducted a four-center case-control study analyzing the relationship between three *XPD* polymorphisms and neuroblastoma risk in the Chinese population.

## RESULTS

### Correlation between *XPD* polymorphisms and neuroblastoma susceptibility

A total of 505 patients and 1070 healthy controls were successfully genotyped in our study. The demographic characteristics of the tested subjects can be found in our previously published articles [36–39] and in Supplemental Table 1. The genotype frequencies for the three selected *XPD* polymorphisms (rs3810366, rs13181, rs238406) in all subjects and in selected subject groups and their contributions to neuroblastoma risk are summarized in Table 1 and Supplemental Table 2. No single *XPD* polymorphism was significantly associated with neuroblastoma risk in any genetic model evaluated. On the other hand, participants harboring 2 or 3 risk genotypes were more likely to develop neuroblastoma (adjusted OR=1.31; 95% CI=1.06-1.62, *P*=0.013) than those with 0 or 1 risk genotype.

**Table 1:** Logistic regression analysis of the correlation between *XPD* polymorphisms and neuroblastoma risk.

Genotype	Cases (N=505)	Controls (N=1070)	*P* ^a^	Crude OR (95% CI)	*P*	Adjusted OR (95% CI) ^b^	*P* ^b^
rs3810366 (HWE=0.143)							
GG	118 (23.37)	285 (26.64)		1.00		1.00	
GC	261 (51.68)	511 (47.76)		1.23 (0.95-1.60)	0.115	1.23 (0.95-1.60)	0.120
CC	126 (24.95)	274 (25.61)		1.11 (0.82-1.50)	0.494	1.11 (0.82-1.50)	0.502
Additive			0.277	1.05 (0.91-1.22)	0.498	1.05 (0.91-1.22)	0.506
Dominant	387 (76.63)	785 (73.36)	0.165	1.19 (0.93-1.52)	0.166	1.19 (0.93-1.52)	0.171
Recessive	379 (75.05)	796 (74.39)	0.780	0.97 (0.76-1.23)	0.781	0.97 (0.76-1.23)	0.778
rs13181 (HWE=0.971)							
TT	424 (83.96)	905 (84.58)		1.00		1.00	
TG	75 (14.85)	158 (14.77)		1.01 (0.75-1.37)	0.931	1.01 (0.75-1.36)	0.943
GG	6 (1.19)	7 (0.65)		1.83 (0.61-5.48)	0.280	1.84 (0.61-5.50)	0.278
Additive			0.548	1.08 (0.83-1.41)	0.586	1.08 (0.82-1.40)	0.594
Dominant	81 (16.04)	165 (15.42)	0.752	1.05 (0.78-1.40)	0.751	1.05 (0.78-1.40)	0.762
Recessive	499 (98.81)	1063 (99.35)	0.274	1.83 (0.61-5.46)	0.281	1.83 (0.61-5.49)	0.279
rs238406 (HWE=0.325)							
GG	133 (26.34)	317 (29.63)		1.00		1.00	
GT	264 (52.28)	516 (48.22)		1.22 (0.95-1.57)	0.121	1.22 (0.95-1.57)	0.119
TT	108 (21.39)	237 (22.15)		1.09 (0.80-1.47)	0.595	1.09 (0.80-1.48)	0.578
Additive			0.282	1.05 (0.91-1.22)	0.508	1.05 (0.91-1.22)	0.492
Dominant	372 (73.66)	753 (70.37)	0.177	1.18 (0.93-1.49)	0.178	1.18 (0.93-1.50)	0.172
Recessive	397 (78.61)	833 (77.85)	0.732	0.96 (0.74-1.24)	0.734	0.96 (0.74-1.24)	0.749
Combined effect of risk genotypes ^c^							
0-1	247 (48.91)	595 (55.61)		1.00		1.00	
2-3	258 (51.09)	475 (44.39)	0.013	**1.31 (1.06-1.62)**	**0.013**	**1.31 (1.06-1.62)**	**0.013**

^a^ *χ^2^* test for genotype distributions between neuroblastoma patients and cancer-free controls. ^b^ Adjusted for age and gender. ^c^ Risk genotypes were rs3810366 GC/GG, rs13181 GG and rs238406 GT/TT.

### Stratification analysis of *XPD* polymorphisms and neuroblastoma susceptibility

To assess the correlations between *XPD* polymorphisms and neuroblastoma risk in particular subgroups of healthy individuals and neuroblastoma patients, we conducted analyses after stratifying based on age, gender, sites of origin, and clinical stages. We found a significant association between the rs3810366 GC/CC genotypes and neuroblastoma risk in participants under age ≤18 months (adjusted OR=1.66, 95% CI=1.10-2.49, *P*=0.015) and in the clinical stage I+II+4s subgroup (adjusted OR=1.50, 95% CI=1.06-1.11, *P*=0.021) (Table 2). After combining the risk genotypes (Table 2), we observed that patients in the following subgroups with 2-3 risk genotypes were more likely to develop a tumor: age ≤18 months (adjusted OR=1.43, 95% CI=1.02-2.02, *P*=0.041), male (adjusted OR=1.33, 95% CI=1.01-1.76, *P*=0.046), tumor originated from others (adjusted OR=2.29, 95% CI=1.20-4.36, *P*=0.012), and clinical stage I+II+4s (adjusted OR=1.53, 95% CI=1.16-2.01, *P*=0.003). We then performed haplotype analysis to determine whether any haplotype carriers were more likely to develop neuroblastoma (Table 3). However, no *XPD* haplotype was associated with neuroblastoma susceptibility when the most common haplotype (GTG) was used as the reference.

**Table 2:** Stratification analysis of associations between *XPD* genotypes and neuroblastoma susceptibility.

Variables	rs3810366 (case/control)		Adjusted OR^a^	*P*^a^	rs13181 (case/control)		Adjusted OR^a^	*P*^a^	rs238406 (case/control)		Adjusted OR^a^	*P*^a^	Risk genotypes (case/control)		Adjusted OR^a^	*P*^a^
	GG	GC/CC	(95% CI)		TT	TG/GG	(95% CI)		GG	GT/TT	(95% CI)		0-1	2-3	(95% CI)	
Age, month																
≤18	40/130	149/295	**1.66 (1.10-2.49)**	**0.015**	156/361	33/64	1.19 (0.75-1.88)	0.464	55/124	134/301	1.00 (0.69-1.46)	0.997	94/249	95/176	**1.43 (1.02-2.02)**	**0.041**
>18	78/155	238/490	0.96 (0.70-1.32)	0.804	268/544	48/101	0.96 (0.66-1.40)	0.829	78/193	238/452	1.31 (0.96-1.78)	0.085	153/346	163/299	1.24 (0.94-1.62)	0.125
Gender																
Female	46/109	167/339	1.17 (0.79-1.73)	0.440	182/373	31/75	0.85 (0.54-1.34)	0.478	61/142	152/306	1.16 (0.81-1.65)	0.427	106/250	107/198	1.27 (0.92-1.77)	0.147
Male	72/176	220/446	1.20 (0.87-1.65)	0.270	242/532	50/90	1.22 (0.84-1.78)	0.305	72/175	220/447	1.20 (0.87-1.65)	0.258	141/345	151/277	**1.33 (1.01-1.76)**	**0.046**
Sites of origin																
Adrenal gland	38/285	135/785	1.27 (0.86-1.86)	0.231	149/905	24/165	0.86 (0.54-1.37)	0.529	48/317	125/753	1.12 (0.78-1.61)	0.534	86/595	87/475	1.28 (0.92-1.76)	0.140
Retroperitoneal	41/285	106/785	0.96 (0.65-1.42)	0.850	126/905	21/165	0.93 (0.57-1.53)	0.785	35/317	112/753	1.32 (0.88-1.98)	0.175	74/595	73/475	1.24 (0.87-1.75)	0.227
Mediastinum	32/285	103/785	1.16 (0.76-1.77)	0.489	107/905	28/165	1.44 (0.92-2.25)	0.113	42/317	93/753	0.94 (0.64-1.39)	0.768	72/595	63/475	1.11 (0.77-1.58)	0.586
Others	7/285	35/785	1.89 (0.83-4.30)	0.132	36/905	6/165	0.92 (0.38-2.22)	0.854	8/317	34/753	1.77 (0.81-3.86)	0.153	15/595	27/475	**2.29 (1.20-4.36)**	**0.012**
Clinical stage																
I+II+4s	49/285	201/785	**1.50 (1.06-2.11)**	**0.021**	215/905	35/165	0.90 (0.61-1.33)	0.589	65/317	185/753	1.20 (0.88-1.64)	0.253	113/595	137/475	**1.53 (1.16-2.01)**	**0.003**
III+IV	62/285	170/785	0.96 (0.70-1.33)	0.823	196/905	36/165	0.98 (0.66-1.46)	0.920	66/317	166/753	1.08 (0.79-1.48)	0.642	126/595	106/475	1.04 (0.78-1.39)	0.768

^a^ Adjusted for age and gender, omitting the corresponding stratification factor.

**Table 3:** Association between inferred *XPD* haplotypes and neuroblastoma susceptibility.

Haplotypes ^a^	Cases (n=1010)	Controls (n=2140)	Crude OR (95% CI)	*P*	Adjusted OR ^b^ (95% CI)	*P* ^b^
GTG	267 (26.44)	553 (25.84)	1.00		1.00	
GTT	203 (20.10)	470 (21.96)	0.90 (0.72-1.12)	0.321	0.90 (0.72-1.12)	0.340
GGG	6 (0.59)	7 (0.33)	1.78 (0.59-5.33)	0.307	1.80 (0.60-5.42)	0.295
GGT	21 (2.08)	51 (2.38)	0.85 (0.50-1.45)	0.555	0.85 (0.50-1.44)	0.544
CTG	230 (22.77)	527 (24.63)	0.90 (0.73-1.12)	0.352	0.91 (0.73-1.12)	0.357
CTT	223 (22.08)	418 (19.53)	1.11 (0.89-1.38)	0.371	1.11 (0.89-1.38)	0.370
CGG	27 (2.67)	63 (2.94)	0.89 (0.55-1.43)	0.622	0.88 (0.55-1.42)	0.602
CGT	33 (3.27)	51 (2.38)	1.34 (0.85-2.13)	0.214	1.35 (0.85-2.14)	0.202

^a^ The haplotype order was rs3810366, rs13181, and rs238406. ^b^ Adjusted for age and gender.

## DISCUSSION

To identify *XPD* polymorphisms influencing neuroblastoma tumorigenesis, we performed a hospital-based case-control study involving a total of 505 neuroblastoma patients and 1070 healthy control subjects. All participants were Chinese. To our knowledge, this study is the first investigation assessing the association between *XPD* polymorphisms and neuroblastoma risk in Chinese children.

*XPD* is located on chromosome 19p13.3 [40] and is composed of 23 exons encoding an evolutionarily conserved ATP-dependent helicase. This helicase is responsible for DNA unwinding and transcription initiation. More than 100 mutations have been mapped in *XPD* [41], which could potentially affect the helicase activity of the encoded protein, thereby impeding normal NER function and leading to increased cancer risk [29,42]. Several *XPD* polymorphisms are reportedly associated with cancer risk [43–45]. Among these, rs1799793 (Asp312Asn) and rs13181 (Lys751Gln) in the *XPD* coding region are the two most widely investigated. The rs13181 polymorphism at codon 751 in exon 23 is a non-synonymous A>C substitution, which results in an amino acid change from Lys to Gln. It has been demonstrated that this Lys751Gln polymorphism could decrease DNA repair capacity [46]. In a study conducted in Poland with 430 patients and 430 controls, Magdalena et al. [47] found that the Gln/Gln genotype of rs13181 is associated with an increased risk of ovarian cancer. In our earlier study investigation of the association between two *XPD* polymorphisms (rs238406 and rs13181) and esophageal squamous cell carcinomas risk, we found that rs238406, but not rs13181, was associated with elevated disease risk [34]. The results are somewhat inconclusive, however, since differing relationships between *XPD* polymorphisms and cancer risk have been reported. These discrepancies may reflect differences in the cancer types, sample sizes, population sources, selection criteria for subjects, and environmental exposures. It is therefore necessary to limit conclusions regarding the contributions of *XPD* polymorphisms to cancer risk to a particular population and cancer type.

Given the critical role of *XPD* polymorphisms in cancer risk and the lack of research on their contributions to neuroblastoma risk, we endeavored to assess the association between three *XPD* polymorphisms and neuroblastoma risk in Chinese children. Unexpectedly, we failed to detect any significant contribution by rs13181 or rs238406 to neuroblastoma risk in the overall analysis or in any of the selected subgroups after stratification. Several factors, including the relatively small sample size, low penetrance of a single polymorphism, and population bias may account for the null association. The etiology of neuroblastoma is complex and subject to heterogenetic influence by a variety of risk factors [15,48]. Although a single *XPD* polymorphism may have limited impact on neuroblastoma risk, it would be expected that the combined effects of several polymorphisms might bring about more significant findings. Indeed, in the present study, we observed that participants with two or more risk genotypes of these functional *XPD* polymorphisms were at significantly higher risk of neuroblastoma than those carrying one or fewer risk genotypes. This trend was also observed in earlier studies by ourselves and others [49,50]. These findings are biologically plausible, probably due to the joint effects of multiple functional polymorphisms.

While this study has its merits, it also has several limitations. First, inherent bias could not be excluded, as all the DNA samples were collected in hospitals. Second, although the sample size for the overall analysis was relatively large, after stratification some subgroups were less than 100, which inevitably diminished the statistical power. Third, the included subjects were restricted to unrelated Han Chinese; consequently, the results may not be applicable to other ethnicities. Fourth, only three *XPD* polymorphisms were selected and analyzed in this study. Other potentially functional *XPD* polymorphisms may also modify the activity of gene or the encoded helicase and thus should be involved in the ongoing study. Fifth, as neuroblastoma is a heterogeneous disease with a complex etiology, the genetic analysis in the present study only partially elucidated the etiology of neuroblastoma. Potentially important environmental factors such as diet, living environment, and parental exposures should be addressed in the future.

In summary, our findings provide insight into the potential role of *XPD* polymorphisms in neuroblastoma risk. Our results failed to detect a role for any single *XPD* polymorphism in neuroblastoma risk. It is anticipated that ongoing epidemiological studies with larger samples and more analysis of confounding factors will provide additional information on the contribution of *XPD* polymorphisms to neuroblastoma tumorigenesis.

## MATERIALS AND METHODS

### Study subjects

A total of 505 cases and 1070 healthy controls were included in this study. Of those, 429 cases and 884 controls were described in our previous study [39]. The additional 76 cases and 186 controls were from the Second Affiliated Hospital of Xi'an Jiaotong University (Supplemental Table 1). The cases were individuals diagnosed with neuroblastoma, and the controls were recruited from the same hospitals between September 2009 and March 2018. The eligibility criteria for the included subjects were described previously [49,51–53]. Written informed consent was provided by all subjects or their guardians. The study protocols were approved by the Institutional Review Board of each hospital.

### Polymorphism selection and genotyping

In brief, we searched for potentially functional candidate SNPs located in the 5’- flanking region, 5’ untranslated region, 3’ untranslated region, and exon of *XPD*. The potentially functional *XPD* polymorphisms were screened from the NCBI dbSNP database (http://www.ncbi.nlm.nih.gov/projects/SNP) and SNPinfo (http://snpinfo.niehs.nih.gov/snpfunc.htm) using previously described criteria [54]. Three polymorphisms (rs3810366 G>C, rs13181 T>G, and rs238406 G>T) in the *XPD* gene were ultimately selected: rs3810366 G>C, which is located within transcription factor binding sites; rs13181 T>G and rs238406 G>T, which may affect splicing regulation activity; and rs13181 T>G, which may also lead to Lys751Gln alteration. As shown in Supplementary Figure 1, there was no significant LD (R^2^<0.8) between rs13181 and rs238406 (R^2^=0.026) or between rs13181 and rs3810366 (R^2^=0.001). However, there was a little LD between rs238406 and rs3810366 (R^2^=0.891).

For genotyping, DNA samples were mainly purified from venous blood using a TIANamp Blood DNA Kit (TianGen Biotech Co. Ltd., Beijing, China). Following standard methods, we used TaqMan real-time PCR to genotype the selected polymorphisms. Details of the genotyping protocol are provided elsewhere [54–57]. To control for result quality, approximately 10% of the samples were randomly selected to perform duplicate analyses. We obtained a concordance rate of 100% for all duplicate sets.

### Statistical analysis

We first used a goodness-of-fit χ^2^ test to assess whether the selected polymorphisms were in Hardy-Weinberg equilibrium in the controls. The demographic variables and allele frequencies were compared between the cases and controls using a two-sided χ^2^ test. The association between *XPD* polymorphisms and neuroblastoma risk was estimated using logistic regression analysis providing odds ratios (ORs) and 95% confidence intervals (CIs). Values of *P*<0.05 were considered significant. All statistical analyses were performed using the SAS statistical package (version 9.1, SAS Institute, Cary, NC).

## SUPPLEMENTARY MATERIAL

Supplemental Table 1

Supplemental Table 2

Supplementary Figure 1
