# Supplementary material for: Relevance of XPD polymorphisms to neuroblastoma risk in Chinese children: a four-center case-control study
Source: Aging (Albany NY). 2018 Aug 8;10(8):1989–2000. doi: 10.18632/aging.101522 (PMC6128416; doi:10.18632/aging.101522)
Supplement: Supplemental Table 1 [file aging-10-101522-s001.docx]

| **Supplemental Table 1. Frequency distribution of selected characteristics in cases and controls.** | | | | | | | | | | |
| --- | --- | --- | --- | --- | --- | --- | --- | --- | --- | --- |
| Variables | Combined subjects | | | | | Shaanxi province | | | | |
|  | Cases (n=505) | | Controls (n=1070) | | *P* ^a^ | Cases (n=76) | | Controls (n=186) | | *P* ^a^ |
|  | No. | % | No. | % |  | No. | % | No. | % |  |
| Age range, months | 0.00-132.00 | | 0.03-156.00 | | 0.384 | 0.07-89.00 | | 0.03-60.00 | | 0.110 |
| Mean ± SD | 33.08±27.38 | | 32.27±26.89 | |  | 24.48±25.23 | | 23.66±16.66 | |  |
| ≤18 | 189 | 37.43 | 425 | 39.72 |  | 43 | 56.58 | 85 | 45.70 |  |
| >18 | 316 | 62.57 | 645 | 60.28 |  | 33 | 43.42 | 101 | 54.30 |  |
| Gender |  |  |  |  | 0.908 |  |  |  |  | 0.778 |
| Female | 213 | 42.18 | 448 | 41.87 |  | 28 | 36.84 | 72 | 38.71 |  |
| Male | 292 | 57.82 | 622 | 58.13 |  | 48 | 63.16 | 114 | 61.29 |  |
| INSS stages |  |  |  |  |  |  |  |  |  |  |
| I | 148 | 29.31 | / | / |  | 64 | 84.21 | / | / |  |
| II | 102 | 20.20 | / | / |  | 7 | 9.21 | / | / |  |
| III | 78 | 15.45 | / | / |  | 1 | 1.32 | / | / |  |
| IV | 154 | 30.50 | / | / |  | 4 | 5.26 | / | / |  |
| 4s | 14 | 2.77 | / | / |  | / | / | / | / |  |
| NA | 9 | 1.78 | / | / |  | / | / | / | / |  |
| Sites of origin |  |  |  |  |  |  |  |  |  |  |
| Adrenal gland | 173 | 34.26 | / | / |  | 9 | 11.84 | / | / |  |
| Retroperitoneal region | 147 | 29.11 | / | / |  | 51 | 67.11 | / | / |  |
| Mediastinum | 135 | 26.73 | / | / |  | 12 | 15.79 | / | / |  |
| Other region | 42 | 8.32 | / | / |  | 4 | 5.26 | / | / |  |
| NA | 8 | 1.58 | / | / |  | / | / | / | / |  |
| SD, standard deviation; NA, not available.  ^a^ Two-sided *χ^2^* test for distributions between neuroblastoma cases and cancer-free controls. | | | | | | | | | | |
