# Supplementary material for: Relevance of XPD polymorphisms to neuroblastoma risk in Chinese children: a four-center case-control study
Source: Aging (Albany NY). 2018 Aug 8;10(8):1989–2000. doi: 10.18632/aging.101522 (PMC6128416; doi:10.18632/aging.101522)
Supplement: Supplemental Table 2 [file aging-10-101522-s002.docx]

| **Supplemental Table 2. Logistic regression analysis for the correlation of *XPD* gene polymorphisms with neuroblastoma risk (Divided subjects).** | | | | | | | | | | | | | | | | |
| --- | --- | --- | --- | --- | --- | --- | --- | --- | --- | --- | --- | --- | --- | --- | --- | --- |
| Genotype | Guangdong province | | | | Henan province | | | | Wenzhou area | | | | Shaanxi Province | | | |
|  | Cases  (N=275) | Controls  (N=531) | AOR (95% CI) ^a^ | *P* ^a^ | Cases  (N=118) | Controls  (N=281) | AOR (95% CI) ^a^ | *P* ^a^ | Cases  (N=36) | Controls  (N=72) | AOR (95% CI) ^a^ | *P* ^a^ | Cases  (N=76) | Controls  (N=186) | AOR (95% CI) ^a^ | *P* ^a^ |
| rs3810366 G>C | | | | | | | | | | | | | | | | |
| GG | 67 (24.36) | 155 (29.19) | 1.00 |  | 27 (22.88) | 63 (22.42) | 1.00 |  | 8 (22.22) | 20 (27.78) | 1.00 |  | 16 (21.05) | 47 (25.27) | 1.00 |  |
| GC | 141 (51.27) | 248 (46.70) | 1.31 (0.92-1.86) | 0.139 | 63 (53.39) | 135 (48.04) | 1.07 (0.62-1.84) | 0.816 | 19 (52.78) | 40 (55.56) | 1.14 (0.42-3.08) | 0.794 | 38 (50.00) | 88 (47.31) | 1.29 (0.65-2.56) | 0.470 |
| CC | 67 (24.36) | 128 (24.11) | 1.21 (0.80-1.83) | 0.358 | 28 (23.73) | 83 (29.54) | 0.78 (0.42-1.45) | 0.422 | 9 (25.00) | 12 (16.67) | 1.85 (0.56-6.13) | 0.315 | 22 (28.95) | 51 (27.42) | 1.31 (0.61-2.84) | 0.490 |
| Additive |  |  | 1.10 (0.90-1.35) | 0.338 |  |  | 0.88 (0.65-1.19) | 0.393 |  |  | 1.35 (0.74-2.49) | 0.328 |  |  | 1.14 (0.78-1.66) | 0.505 |
| Dominant | 208 (75.64) | 376 (70.81) | 1.28 (0.91-1.78) | 0.154 | 91 (77.12) | 218 (77.58) | 0.96 (0.57-1.60) | 0.862 | 28 (77.78) | 52 (72.22) | 1.30 (0.51-3.36) | 0.582 | 60 (78.95) | 139 (74.73) | 1.30 (0.68-2.48) | 0.434 |
| Recessive | 208 (75.64) | 403 (75.89) | 1.02 (0.73-1.43) | 0.906 | 90 (76.27) | 198 (70.46) | 0.74 (0.45-1.22) | 0.236 | 27 (75.00) | 60 (83.33) | 1.69 (0.63-4.52) | 0.296 | 54 (71.05) | 135 (72.58) | 1.10 (0.60-2.01) | 0.753 |
| rs13181 T>G | | | | | | | | | | | | | | | | |
| TT | 230 (83.64) | 462 (87.01) | 1.00 |  | 97 (82.20) | 230 (81.85) | 1.00 |  | 30 (83.33) | 59 (81.94) | 1.00 |  | 67 (88.16) | 154 (82.80) | 1.00 |  |
| TG | 41 (14.91) | 65 (12.24) | 1.29 (0.84-1.96) | 0.245 | 19 (16.10) | 50 (17.79) | 0.88 (0.49-1.59) | 0.680 | 6 (16.67) | 12 (16.67) | 1.03 (0.35-3.07) | 0.954 | 9 (11.84) | 31 (16.67) | 0.67 (0.30-1.48) | 0.321 |
| GG | 4 (1.45) | 4 (0.75) | 2.00 (0.50-8.11) | 0.331 | 2 (1.69) | 1 (0.36) | 4.72 (0.42-53.11) | 0.209 | 0 (0.00) | 1 (1.39) | / | / | 0 (0.00) | 1 (0.54) | / | / |
| Additive |  |  | 1.32 (0.91-1.90) | 0.142 |  |  | 1.04 (0.62-1.77) | 0.874 |  |  | 0.87 (0.32-2.36) | 0.779 |  |  | 0.64 (0.29-1.39) | 0.256 |
| Dominant | 45 (16.36) | 69 (12.99) | 1.33 (0.88-2.00) | 0.175 | 21 (17.80) | 51 (18.15) | 0.96 (0.54-1.69) | 0.881 | 6 (16.67) | 13 (18.06) | 0.94 (0.32-2.75) | 0.910 | 9 (11.84) | 32 (17.20) | 0.65 (0.29-1.43) | 0.284 |
| Recessive | 271 (98.55) | 524 (99.25) | 1.93 (0.48-7.82) | 0.355 | 116 (98.31) | 280 (99.64) | 4.84 (0.43-54.27) | 0.202 | 36 (100.00) | 71 (98.61) | / | / | 76 (100.00) | 185 (99.46) | / | / |
| rs238406 G>T | | | | | | | | | | | | | | | | |
| GG | 73 (26.55) | 149 (28.06) | 1.00 |  | 32 (27.12) | 96 (34.16) | 1.00 |  | 12 (33.33) | 15 (20.83) | 1.00 |  | 16 (21.05) | 57 (30.65) | 1.00 |  |
| GT | 143 (52.00) | 250 (47.08) | 1.16 (0.82-1.65) | 0.395 | 61 (51.69) | 131 (46.62) | 1.41 (0.85-2.33) | 0.182 | 17 (47.22) | 39 (54.17) | 0.54 (0.21-1.41) | 0.212 | 43 (56.58) | 96 (51.61) | 1.59 (0.82-3.10) | 0.174 |
| TT | 59 (21.45) | 132 (24.86) | 0.91 (0.60-1.38) | 0.654 | 25 (21.19) | 54 (19.22) | 1.44 (0.77-2.70) | 0.251 | 7 (19.44) | 18 (25.00) | 0.51 (0.16-1.63) | 0.252 | 17 (22.37) | 33 (17.74) | 1.83 (0.81-4.17) | 0.149 |
| Additive |  |  | 0.96 (0.78-1.18) | 0.710 |  |  | 1.22 (0.90-1.65) | 0.207 |  |  | 0.70 (0.38-1.26) | 0.231 |  |  | 1.36 (0.91-2.04) | 0.135 |
| Dominant | 202 (73.45) | 382 (71.94) | 1.08 (0.78-1.49) | 0.664 | 86 (72.88) | 185 (65.84) | 1.42 (0.88-2.29) | 0.151 | 24 (66.67) | 57 (79.17) | 0.53 (0.22-1.31) | 0.171 | 60 (78.95) | 129 (69.35) | 1.65 (0.87-3.13) | 0.127 |
| Recessive | 216 (78.55) | 399 (75.14) | 0.83 (0.58-1.17) | 0.280 | 93 (78.81) | 227 (80.78) | 1.17 (0.68-1.99) | 0.576 | 29 (80.56) | 54 (75.00) | 0.76 (0.28-2.04) | 0.578 | 59 (77.63) | 153 (82.26) | 1.33 (0.68-2.59) | 0.406 |
| Combined effect of risk genotypes ^b^ | | | | | | | | | | | | | | | | |
| 0-1 | 137 (49.82) | 300 (56.50) | 1.00 |  | 58 (49.15) | 158 (56.23) | 1.00 |  | 20 (55.56) | 34 (47.22) | 1.00 |  | 32 (42.11) | 103 (55.38) | 1.00 |  |
| 2-3 | 138 (50.18) | 231 (43.50) | 1.30 (0.97-1.74) | 0.079 | 60 (50.85) | 123 (43.77) | 1.33 (0.86-2.05) | 0.195 | 16 (44.44) | 38 (52.78) | 0.70 (0.31-1.58) | 0.391 | 44 (57.89) | 83 (44.62) | 1.71 (1.00-2.93) | 0.052 |
| ^a^ Adjusted for age and gender.  ^b^ Risk genotypes were rs3810366 GC/GG, rs13181 GG and rs238406 GT/TT. | | | | | | | | | | | | | | | | |
