# Supplementary material for: Relevance of XPD polymorphisms to neuroblastoma risk in Chinese children: a four-center case-control study
Source: Aging (Albany NY). 2018 Aug 8;10(8):1989–2000. doi: 10.18632/aging.101522 (PMC6128416; doi:10.18632/aging.101522)

**Supplementary Figure 1**. **Linkage disequilibrium (LD) analysis for the three selected SNPs in Chinese Han population consisting of CHB (Han Chinese in Beijing, China) and CHS (Southern Han Chinese) subjects**. LD as R^2^ for SNP pairs is shown inside the squares.


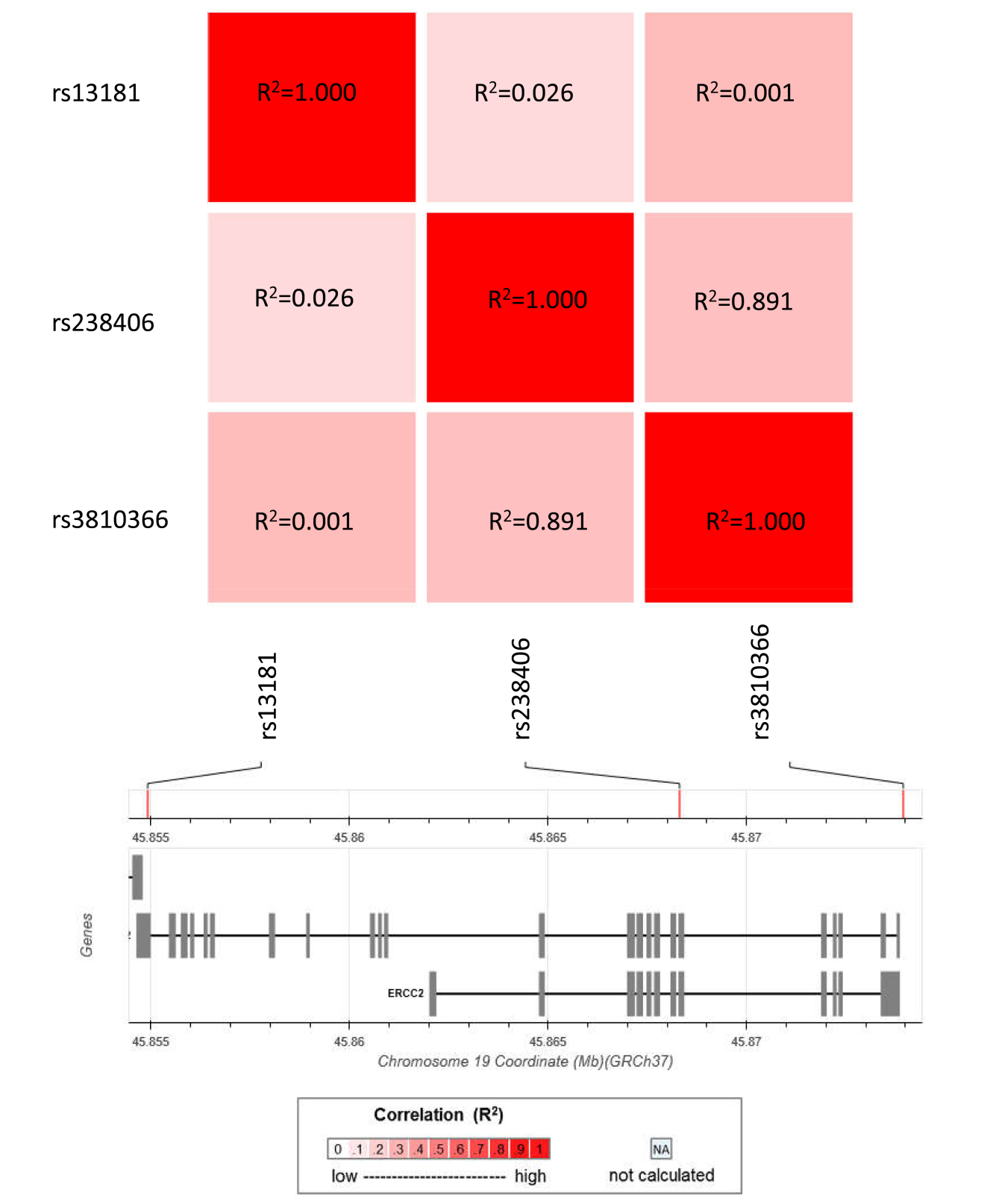

Supplement: Supplementary Figure 1 [file aging-10-101522-s003.docx]
